# The admission pH is a risk factor of preoperative deep vein thrombosis in geriatric hip fracture: a retrospective cohort study

**DOI:** 10.1038/s41598-023-45712-0

**Published:** 2023-10-26

**Authors:** Bao-Hui Wang, Yin-Di Sun, Xiao-Chen Fan, Bin-Fei Zhang

**Affiliations:** 1 Pain Area of Rehabilitation Hospital, Honghui Hospital, Xi’an Jiaotong University, Xi’an, Shaanxi Province China; 2 Department of Joint Surgery, Honghui Hospital, Xi’an Jiaotong University, No. 555 Youyi East Road, Xi’an, 710054 Shaanxi Province China

**Keywords:** Ecology, Genetics, Biomarkers, Diseases, Health care, Risk factors

## Abstract

This study evaluated the association between body pH value and preoperative deep vein thrombosis (DVT) in geriatric hip fractures. Older adult patients with hip fractures were screened between January 2015 and September 2019. The demographic and clinical characteristics of the patients were collected. Multivariate binary logistic regression and generalized additive models were used to identify the linear and nonlinear associations between pH value and preoperative DVT. Analyses were performed using EmpowerStats and R software. A total of 1465 patients were included in the study. DVT occurred in 476 (32.6%) of these admitted older adults. We observed a nonlinear association between the serum pH value and preoperative DVT in geriatric patients with hip fractures. A pH value of 7.39 was the inflection point in the curve, with pH highly correlated with DVT at pH < 7.39 (odds ratio [OR] 19.47; 95% confidence interval [CI] 1.45–260.91; *P* = 0.0249). Patients with lower pH had a lower chance of preoperative DVT formation, and the risk of DVT increased 18.47-fold for every 0.1 unit change in pH. Although at pH > 7.39, pH was not correlated with DVT (OR 1.26; 95% CI 0.85–1.86; *P* = 0.2561), the odds of DVT did not vary with pH, and the highest risk of thrombosis was reached. The body pH value is nonlinearly associated with preoperative DVT in geriatric patients with hip fractures, and it could be considered a predictor of the risk of DVT.

*Registered information* This study is registered in the website of Chinese Clinical Trial Registry (ChiCTR: ChiCTR2200057323).

## Introduction

With the progression of the aging population, hip fractures in the elderly have become a severe public health problem worldwide^1^. Deep vein thrombosis (DVT) is a serious complication of hip fractures during hospitalization and after discharge. The incidence of DVT after hip fracture in the elderly is high^2^. Studies have found a preoperative DVT rate of up to 32% in patients with hip fractures^3^. Even with routine anticoagulation prophylaxis, the incidence of symptomatic DVT in patients with hip fractures remains as high as 1.3%–10% 3 months after surgery^4^. The incidence of DVT screening in patients with hip fracture immediately after admission can still range from 20.10% to 31.36%, and 21.93% of elderly patients after hip fracture develop lower limb DVT within 24 h of injury^5,6^. DVT, a form of venous thromboembolism, can cause secondary pulmonary thromboembolism in patients who develop DVT, with a mortality rate of approximately 4%. Post-thrombotic syndrome caused by DVT significantly affects patients' quality of life and can even lead to death^7,8^. This group of patients should be given high priority in clinical practice. The insidious nature of DVT makes it easy to overlook, which delays the implementation of prevention, early diagnosis, and treatment^9^. The early warning and diagnosis of DVT are of great clinical importance and value. DVT is believed to be caused by a combination of genetic, environmental, and behavioral factors, with 50%–60% of its occurrence attributable to genetic factors^10^.

In addition, it has been reported that acid–base balance, known as pH, is a regulatory mechanism by which the pH of extracellular fluid is stringently maintained^11^. The intracellular pH is an essential aspect of the intracellular environment. Changes in intracellular pH can affect virtually all cellular processes, including metabolism, membrane potential, cell growth, movement of substances across the surface membrane, state of polymerization of the cytoskeleton, and the ability to contract in muscle cells. Changes in intracellular pH are often a response of cells to externally applied agents^12^. Alterations in pH could lead to DVT^13,14^. The pH value of normal human blood is 7.35–7.45, and solutions outside this range damage vascular endothelial cells and induce platelet aggregation^15^. In addition, changes in pH will affect the water absorption capacity of vein intima epithelial cells, leading to more significant changes in the permeability of blood vessels, such as local redness and swelling of blood vessels, adverse effects on human blood circulation, and decreased metabolic function of vascular intima, which will ultimately affect blood coagulation function^16,17^.

We hypothesized that pH may be associated with DVT formation in hip fractures. However, the relationship between admission pH and preoperative DVT in patients with hip fractures remains unclear. Therefore, this study aimed to assess the association between admission pH and preoperative DVT through long-term follow-up in an elderly population.

## Materials and methods

### Study design

In this retrospective cohort study, we recruited older adults who had a hip fracture from January 1, 2015, to September 30, 2019, at the largest trauma center in Northwest China.

This retrospective study was approved by the Ethics Committee of Xi’an Honghui Hospital (No. 202201009). Informed consent was obtained from all patients. All human procedures were performed following the 1964 Declaration of Helsinki and its later amendments. The study was conducted according to the STROCSS 2021 guidelines^18^.

### Participants

The demographic and clinical data of the patients were obtained from their original medical records. The inclusion criteria were as follows: (1) age of ≥ 65 years; (2) X-ray or computed tomography diagnosis of the femoral neck, intertrochanteric, or subtrochanteric fracture; (3) patients who were receiving surgical or conservative treatment in the hospital; and (4) availability of clinical data in the hospital. Exclusion criteria: patients who did not receive the pharmacological thromboprophylaxis because of contraindications.

### Hospital treatment

Patients were examined using blood tests to prepare for surgery. Prophylaxis for DVT was initiated on admission. Mechanical thromboprophylaxis (foot intermittent pneumatic compression sleeve, 20 min twice daily) was used to prevent DVT. In patients without contraindications, low-molecular-weight heparin was injected subcutaneously to prevent DVT. Doppler ultrasonography was performed to diagnose DVT by two senior sonographers (Hong Zhang, Yi-Lun Wu). The diagnostic criterion was the presence of a constant intraluminal filling defect. All patients were examined preoperatively. All patients underwent ultrasonography of the bilateral lower extremities the day before the scheduled surgery.

### Endpoint events

The endpoint event in this study was DVT before operation.

### Variables

The variables collected in this study were as follows: age, sex, occupation, history of allergy, injury mechanism, fracture classification, hypertension, diabetes, coronary heart disease, arrhythmia, hemorrhagic stroke, ischemic stroke, cancer, associated injuries, dementia, chronic obstructive pulmonary disease, hepatitis, gastritis, age-adjusted Charlson comorbidity index (aCCI), time from injury to admission, blood predictors and pH value.

The dependent variable was preoperative DVT, and the independent variable was the pH value. The other variables were potentially confounding factors.

### Statistics analysis

Continuous variables were reported as mean ± standard deviation (Gaussian distribution) or median (minimum and maximum) (skewed distribution), and categorical variables were presented as frequencies and percentages. We used χ2 (categorical variables), one-way ANOVA (normal distribution), or the Kruskal–Wallis H test (skewed distribution) to test for differences among different pH values (quartile). Using three distinct models, we used univariate and multivariate binary logistic regression models to test the association between pH and preoperative DVT. Model 1 was a non-adjusted model with no covariates adjusted for. Model 2 was a minimally adjusted model with adjusted sociodemographic variables. Model 3 was a fully adjusted model with the covariates presented in Table 1. To account for the nonlinear relationship between pH and preoperative DVT, we also used a generalized additive model and smooth curve fitting (penalized spline method) to address nonlinearity. In addition, a two-piecewise binary logistic regression model was used to explain nonlinearity further. To test the robustness of our results, we performed a sensitivity analysis. We converted pH into a categorical variable according to the quartile. We calculated the *P* for the trend to verify the results of pH as a continuous variable and examine the possibility of nonlinearity. In addition, propensity score matching (PSM) was introduced for matched groups and adjusted for confounding factors in the PSM models.

**Table 1:** The demographic and clinical characteristics.

pH tertiles	Low	Middle	High	*P *value	*P* value*
N	365	531	569		
Age (year)	79.64 ± 6.74	79.71 ± 6.84	80.07 ± 6.78	0.554	0.673
pH value	7.39 ± 0.02	7.43 ± 0.01	7.47 ± 0.02	< 0.001	< 0.001
Sex				0.314	–
Male	119 (32.60%)	157 (29.57%)	159 (27.94%)		
Female	246 (67.40%)	374 (70.43%)	410 (72.06%)		
Injury mechanism				0.071	–
Falling	350 (95.89%)	515 (96.99%)	550 (96.66%)		
Accident	15 (4.11%)	13 (2.45%)	12 (2.11%)		
Other	0 (0.00%)	3 (0.56%)	7 (1.23%)		
Fracture classification				0.148	–
Intertrochanteric fracture	219 (60.00%)	331 (62.34%)	318 (55.89%)		
Femoral neck fracture	137 (37.53%)	194 (36.53%)	239 (42.00%)		
Subtrochanteric fracture	9 (2.47%)	6 (1.13%)	12 (2.11%)		
Hypertension	170 (46.58%)	253 (47.65%)	292 (51.32%)	0.294	–
Diabetes	92 (25.21%)	85 (16.01%)	98 (17.22%)	0.001	–
CHD	174 (47.67%)	255 (48.02%)	308 (54.13%)	0.066	–
Arrhythmia	115 (31.51%)	153 (28.81%)	187 (32.86%)	0.341	–
Hemorrhagic stroke	3 (0.82%)	8 (1.51%)	11 (1.93%)	0.395	–
Ischemic stroke	106 (29.04%)	141 (26.55%)	204 (35.85%)	0.003	–
Cancer	5 (1.37%)	21 (3.95%)	11 (1.93%)	0.027	–
Multiple injuries	24 (6.58%)	38 (7.16%)	42 (7.38%)	0.894	–
Dementia	8 (2.19%)	18 (3.39%)	31 (5.45%)	0.032	–
COPD	30 (8.22%)	29 (5.46%)	28 (4.92%)	0.097	–
Hepatitis	8 (2.19%)	15 (2.82%)	22 (3.87%)	0.322	–
Gastritis	5 (1.37%)	11 (2.07%)	7 (1.23%)	0.501	–
aCCI	4.25 ± 1.10	4.14 ± 1.10	4.32 ± 1.09	0.023	0.029
Time to operation (d)	4 (1–20)	4 (1–34)	4 (1–18)	0.037	0.132
Time to admission (h)	8 (1–5040)	10 (1–2880)	24 (1–1440)	0.714	< 0.001
Glomerular filtration rate (ml/min)	71.96 ± 21.07	77.22 ± 18.14	79.96 ± 15.17	< 0.001	< 0.001
Urea (mmol/L)	7.00 ± 3.08	6.83 ± 2.81	6.38 ± 2.41	0.001	0.003
Creatinine (umol/L)	76.17 ± 36.03	68.83 ± 23.84	65.12 ± 20.41	< 0.001	< 0.001
Cystatin C (mg/L)	0.89 ± 0.38	0.83 ± 0.30	0.80 ± 0.25	< 0.001	0.021
Prothrombin time (s)	12.74 ± 1.41	12.74 ± 1.70	12.85 ± 1.01	0.348	0.006
INR	1.08 ± 0.24	1.08 ± 0.16	1.08 ± 0.10	0.721	0.072
Activated partial thromboplastin time (s)	30.02 ± 5.32	29.80 ± 5.03	30.15 ± 4.66	0.501	0.185
Thrombin time (s)	17.23 ± 3.69	16.93 ± 1.93	16.61 ± 1.30	< 0.001	< 0.001
DVT	107 (29.32%)	171 (32.20%)	198 (34.80%)	0.214	–

Mean + standard deviation/Median (min–max)/N(%)*. P* value*: For continuous variables, we used the Kruskal–Wallis rank-sum test and Fisher’s exact probability test for count variables with a theoretical number of < 10.

All analyses were performed using statistical software packages R (http://www.R-project.org, R Foundation) and EmpowerStats (http://www.empowerstats.com, X&Y Solutions Inc., Boston, MA, USA). Odds ratios (OR) and 95% CIs were calculated. Statistical significance was set at a two-sided *P* value of < 0.05 (two-sided).

### Ethics approval and consent to participate

The study was approved by the Ethics Committee of the Honghui Hospital, Xi’an Jiaotong University (No. 202201009).

### Consent to publish

 The work described has not been published before (except in the form of an abstract or as part of a published lecture, review, or thesis); it is not under consideration for publication elsewhere; and its publication has been approved by all co-authors.

## Results

### Patient characteristics

In this retrospective cohort study, we screened older adults who had hip fractures in the hospital from January 1, 2015, to September 30, 2019, and recruited 1470 study participants who met the inclusion criteria. Five patients were excluded because of pharmacological thromboprophylaxis contraindications according to exclusion criteria.

A total of 1465 patients were included in the study. Preoperative DVT occurred in 476 (32.6%) admitted older adults. Table 1 lists the demographic and clinical characteristics of the study population after dividing the pH tertiles into the low, middle, and high groups.

### Univariate analysis of the association between variates and DVT

We performed a univariate analysis to identify the relationship between the study factors and DVT. The results in Table 2 show differences in DVT according to sex, fracture classification, multiple injuries, dementia, gastritis, time to operation, and pH. To more accurately investigate the relationship between pH and DVT, confounding factors were identified at *P* < 0.1. Sex, fracture classification, multiple injuries, time to operation, dementia, gastritis, and activated partial thromboplastin time were identified as confounding factors.

**Table 2:** Effects of factors on DVT measured by univariate analysis.

	Statistics	OR (95%CI)	*P* value
Age (year)	79.83 ± 6.79	1.00 (0.98, 1.01)	0.5781
Sex			
Male	435 (29.69%)	1	
Female	1030 (70.31%)	1.30 (1.02, 1.66)	0.0345
Injury mechanism			
Falling	1415 (96.59%)	1	
Accident	40 (2.73%)	1.26 (0.66, 2.42)	0.483
Other	10 (0.68%)	2.10 (0.61, 7.30)	0.2417
Fracture classification			
Intertrochanteric fracture	868 (59.25%)	1	
Femoral neck fracture	570 (38.91%)	0.71 (0.56, 0.89)	0.0035
Subtrochanteric fracture	27 (1.84%)	2.71 (1.24, 5.92)	0.0122
Hypertension	715 (48.81%)	1.10 (0.88, 1.37)	0.3911
Diabetes	275 (18.77%)	0.84 (0.63, 1.12)	0.2332
CHD	737 (50.31%)	1.17 (0.94, 1.46)	0.162
Arrhythmia	455 (31.06%)	1.19 (0.94, 1.51)	0.1427
Hemorrhagic stroke	22 (1.50%)	1.75 (0.75, 4.07)	0.1963
Ischemic stroke	451 (30.78%)	0.88 (0.69, 1.12)	0.3024
Cancer	37 (2.53%)	1.27 (0.65, 2.50)	0.4828
Multiple injuries	104 (7.10%)	1.65 (1.10, 2.47)	0.0157
Dementia	57 (3.89%)	1.92 (1.13, 3.27)	0.016
COPD	87 (5.94%)	0.93 (0.58, 1.49)	0.7648
Hepatitis	45 (3.07%)	0.75 (0.38, 1.46)	0.3983
Gastritis	23 (1.57%)	0.19 (0.05, 0.83)	0.0273
aCCI	4.24 ± 1.10	0.96 (0.87, 1.06)	0.3859
Time to admission (h)	84.89 ± 249.52	1.00 (1.00, 1.00)	0.6382
Time to operation (d)	4.17 ± 2.44	1.06 (1.02, 1.11)	0.0081
Glomerular filtration rate (ml/min)	77.12 ± 18.00	1.00 (0.99, 1.01)	0.8435
Urea (mmol/L)	6.70 ± 2.74	1.03 (0.99, 1.07)	0.1674
Creatinine (umol/L)	69.22 ± 26.62	1.00 (1.00, 1.00)	0.971
Cystatin C (mg/L)	0.830.31	1.07 (0.75, 1.52)	0.726
Prothrombin time (s)	12.78 ± 1.39	0.98 (0.90, 1.06)	0.5865
INR	1.08 ± 0.17	0.85 (0.42, 1.73)	0.6598
Activated partial thromboplastin time (s)	29.99 ± 4.96	0.98 (0.96, 1.00)	0.0782
Thrombin time (s)	16.88 ± 2.34	0.94 (0.88, 1.01)	0.1161
pH	74.36 ± 0.34	1.59 (1.15, 2.20)	0.0046

### Multivariate analysis between preoperative pH and DVT

To demonstrate the relationship between pH and DVT in patients, data in Table 3 were further adjusted for confounding factors to perform multifactorial logistic regression. In addition, pH was tested for trend as a categorical variable (in three groups by trichotomization) and as a continuous variable. The results were statistically significant when pH was used as a continuous variable. In contrast, there was no statistical difference between the two results when divided into three groups and in the trend test calculated as a continuous variable. Therefore, the results were unstable, and other relationships were considered possible, most often curvilinear.

**Table 3:** The multivariate results by linear regression.

Exposure	Non-adjusted model	Minimally-adjusted model	Fully-adjusted model
pH	1.59 (1.15, 2.20) 0.0046	1.58 (1.14, 2.18) 0.0054	1.54 (1.09, 2.17) 0.0144
pH tertiles			
Low	Ref	Ref	Ref
Middle	1.15 (0.86, 1.53) 0.3586	1.14 (0.85, 1.52) 0.3857	1.11 (0.82, 1.51) 0.4961
High	1.29 (0.97, 1.71) 0.0816	1.27 (0.96, 1.69) 0.0965	1.25 (0.93, 1.69) 0.1424
*P* for trend	0.0797	0.0941	0.1358

Data in the table: OR (95% CI) *P* value.

Outcome variable: deep vein thrombosis. Exposed variables pH.

The minimally adjusted model was adjusted for sex.

The fully adjusted model was adjusted for sex, fracture classification, multiple injuries, time to operation, dementia, gastritis and activated partial thromboplastin time.

### Curve fitting and analysis of threshold effect

**Figure 1:**
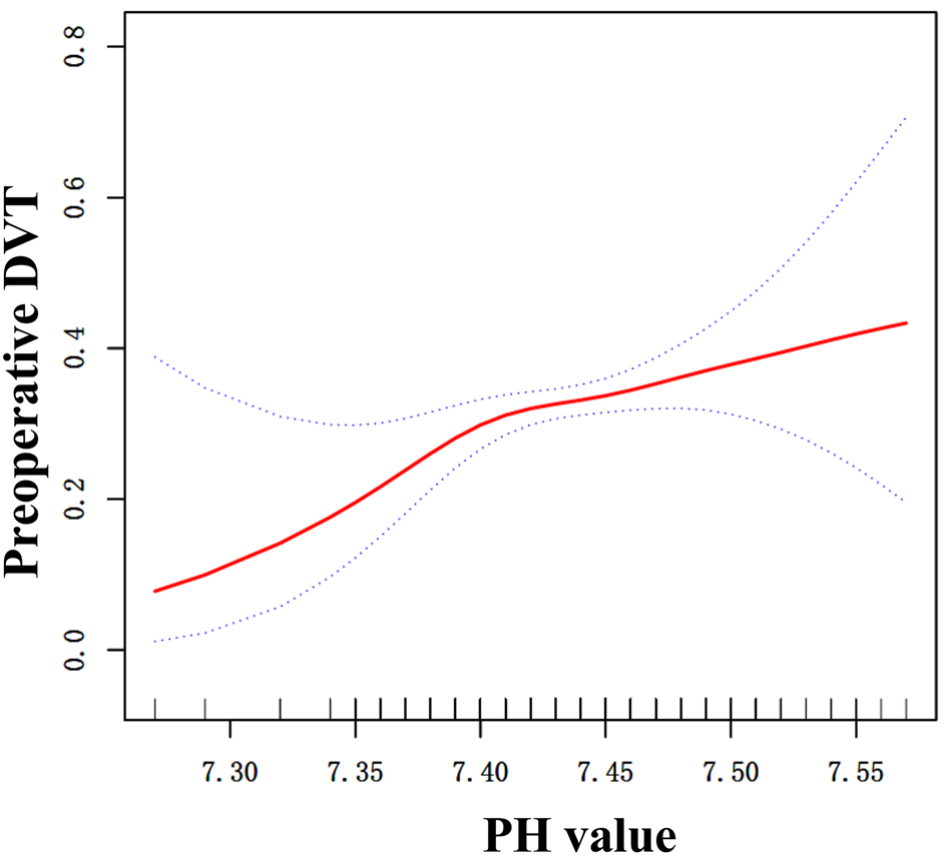
Curve fitting between preoperative pH and deep vein thrombosis (DVT). Adjusted for sex, fracture classification, multiple injuries, time to operation, dementia, gastritis and activated partial thromboplastin time.

A curve fit in Fig. 1 revealed a possible curvilinear relationship; therefore, an attempt was made to identify the curvilinear relationship using Table 4. The result was that there was indeed a curvilinear relationship, that is, the curvilinear relationship held in the current study. A pH value of 7.39 was the inflection point in the curve, and at pH < 7.39, pH was highly correlated with DVT (OR 19.47; 95% CI 1.45–260.91; P = 0.0249), whereas at pH > 7.39, pH was not associated with DVT (OR 1.26; 95% CI 0.85–1.86; *P* = 0.2561).

**Table 4:** Nonlinearity of preoperative pH and deep vein thrombosis.

Outcome:	OR (95%CI) *P *value
Fitting model by stand linear regression	1.54 (1.09, 2.17) 0.0144
Fitting model by two-piecewise linear regression	
Inflection point	7.39
< 7.39	19.47 (1.45, 260.91) 0.0249
> 7.39	1.26 (0.85, 1.86) 0.2561
*P* for log-likelihood ratio test	0.028

Adjusted for sex, fracture classification, multiple injuries, time to operation, dementia, gastritis and activated partial thromboplastin time.

### Propensity score matching

**Figure 2:**
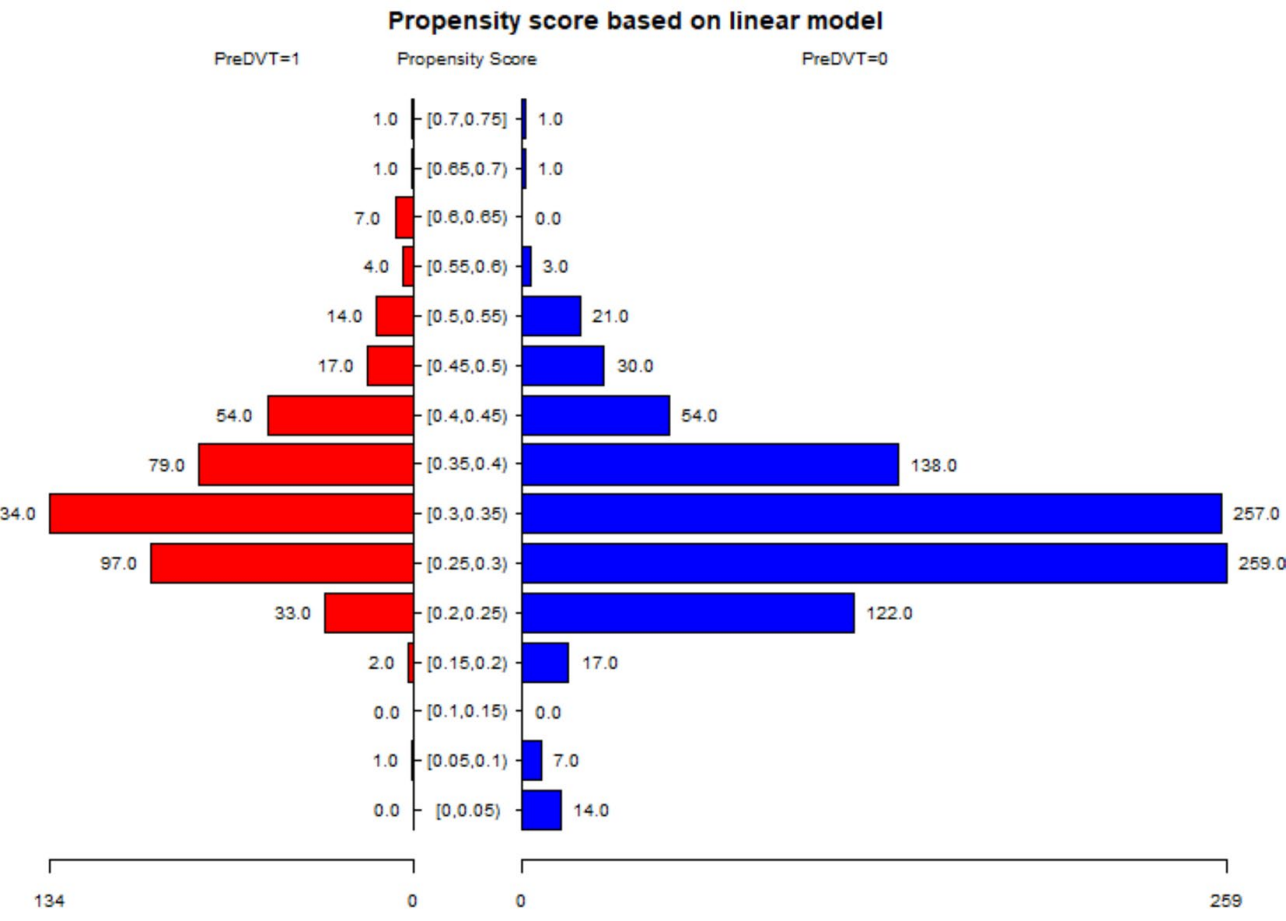
Propensity score matching of two groups under propensity score based on the logistic regression model.

To test the robustness of our results, we performed sensitivity analysis using PSM, as shown in Fig. 2 and Tables 5–7. Eight hundred eighty patients (60.06%) were successfully matched (Fig. 2 and Table 5). Sex, fracture classification, diabetes, hemorrhagic stroke, ischemic stroke, multiple injuries, time to operation, and aCCI did not match between the two groups (Table 6). We found that the results were stable in the multivariate regression results under the PSM and PSM-adjusted models (Table 7). The pH value was 7.39, the curve's inflection point.

**Table 5:** Propensity score parameter list.

The variables used in calculating the propensity score	Age; Sex; Time to operation; Injury mechanism; Time to admission; Fracture classification; Hypertension; Diabetes; CHD; Arrhythmia; Hemorrhagic stroke; Ischemic stroke; Cancer; Multiple injuries; Dementia; COPD; Hepatitis; Gastritis; aCCI
Propensity score algorithm	Logistic regression model
C-statistical	0.6259
Matching method	Greedy matching within specified caliper distances
Metric Distances	0.05
Matching ratio	1:1
Use of replacement	With replacement
Matching sample size	No. of DVT = 1: 440 cases No. of NO DVT = 0: 440 cases Total 880 cases

**Table 6:** The balance test of propensity score matching (N = 880).

Variables	DVT: No (n = 440)	DVT: Yes (n = 440)	Standardized diff	*P* value
Age (year)	80.24 ± 6.82	79.55 ± 6.48	0.1042	0.1227
Sex			0.3007	< 0.0001*
Male	171 (38.9)	110 (25)		
Female	269 (61.1)	330 (75)		
Occupation				0.3786
Retirement	261 (59.3)	245 (55.7)	0.0736	
Farmer	106 (24.1)	124 (28.2)	0.0932	
Other	73 (16.6)	71 (16.1)	0.0123	
History of allergy			0.0552	0.5123
No	423 (96.1)	418 (95)		
Yes	17 (3.9)	22 (5)		
Injury mechanism				0.0939
Falling	432 (98.2)	423 (96.1)	0.1234	
Accident	8 (1.8)	14 (3.2)	0.0874	
Other	0 (0)	3 (0.7)	0.1172	
Fracture classification				0.0003*
Intertrochanteric fracture	227 (51.6)	279 (63.4)	0.2408	
Femoral neck fracture	205 (46.6)	147 (33.4)	0.2715	
Subtrochanteric fracture	8 (1.8)	14 (3.2)	0.0874	
Hypertension	208 (47.3)	222 (50.5)	0.0637	0.3807
Diabetes	112 (25.5)	75 (17)	0.2067	0.003*
CHD	201 (45.7)	225 (51.1)	0.1093	0.1208
Arrhythmia	129 (29.3)	137 (31.1)	0.0396	0.6074
Hemorrhagic stroke	1 (0.2)	9 (2)	0.1722	0.026*
Ischemic stroke	152 (34.5)	124 (28.2)	0.1375	0.0498*
Cancer	13 (3)	11 (2.5)	0.0279	0.836
Multiple injuries	17 (3.9)	39 (8.9)	0.2059	0.0037*
Dementia	12 (2.7)	24 (5.5)	0.138	0.0612
COPD	32 (7.3)	23 (5.2)	0.0846	0.2652
Hepatitis	21 (4.8)	12 (2.7)	0.1078	0.1558
Gastritis	1 (0.2)	1 (0.2)	0	1
aCCI				0.0024*
2	16 (3.6)	16 (3.6)	0	
3	68 (15.5)	97 (22)	0.1695	
4	166 (37.7)	184 (41.8)	0.0837	
5	119 (27)	104 (23.6)	0.0784	
6	58 (13.2)	25 (5.7)	0.2587	
7	11 (2.5)	11 (2.5)	0	
8	2 (0.5)	3 (0.7)	0.0302	
Time to operation (d)	3.86 ± 2.52	4.37 ± 2.47	0.2011	0.0029*
Time to admission (h)	75.10 ± 168.21	87.41 ± 192.83	0.0681	0.313

*Variables were not successfully matched.

**Table 7:** Multivariate results by the regression analysis.

Outcome:	Fully-adjusted model	PSM model	PSM-adjusted model
Fitting model by stand linear regression	1.54 (1.09, 2.17) 0.0144	1.77 (1.20, 2.62) 0.0040	1.89 (1.23, 2.90) 0.0035
Fitting model by two-piecewise linear regression			
Inflection point	7.39	7.39	7.39
< Inflection point	19.47 (1.45, 260.91) 0.0249	33.55 (2.23, 504.16) 0.0111	36.34 (2.08, 633.39) 0.0137
> Inflection point	1.26 (0.85, 1.86) 0.2561	1.35 (0.87, 2.11) 0.1838	1.43 (0.87, 2.33) 0.1563
*P* for log-likelihood ratio test	0.028	0.014	0.021

Data in the table: OR (95% CI) *P* value.

Outcome variable: deep vein thrombosis.

Exposed variables: pH.

Adjusted variables in the propensity score matching (PSM)-adjusted model: sex, fracture classification, diabetes, hemorrhagic stroke, ischemic stroke, multiple injuries, and time to operation, and aCCI.

## Discussion

We observed a linear association between serum pH value and preoperative DVT in geriatric patients with hip fractures. pH is a risk factor for DVT formation; however, its relationship is curvilinear. A pH value of 7.39 was the inflection point in the curve, with pH highly correlated with DVT at pH < 7.39 (OR 19.47; 95% CI 1.45–260.91; *P* = 0.0249). Patients with lower pH had a lower chance of preoperative DVT formation, and the risk of DVT increased 18.47-fold for every 0.1 unit change in pH. Although at pH > 7.39, pH was not correlated with DVT (OR 1.26; 95% CI 0.85–1.86; *P* = 0.2561), the odds of DVT did not vary with pH, and the highest risk of thrombosis was reached. According to the comparison of logistic regression and PSM results, the logistic regression was more accurate^19^ in this study. Indeed, the results of logistic regression and PSM results have the same inflection point in this study.

The accepted pathogenesis of lower limb DVT is based on the three main elements of DVT formation proposed by Virchow in 1856: slow blood flow, intimal damage, and hypercoagulable blood^20^. Although most hip fractures in the elderly are caused by low-energy injuries, with high-energy injuries accounting for a relatively low percentage of injuries^21^, hip injuries have a more significant impact on the systemic condition of elderly patients, especially in terms of hemodynamic instability and imbalance in the homeostasis of the internal environment, which is more likely to stimulate exogenous coagulation mechanisms and increase the risk of DVT formation^22^.

DVT formation is a complex and dynamic physiological process, and susceptibility to DVT thromboembolism is associated with its metabolites^23^. The most commonly described metabolites possibly related to DVT are carnitine species, glucose, phenylalanine, 3-hydroxybutyrate, lactic acid, tryptophan, and some monounsaturated and polyunsaturated fatty acids^14^. Studies have shown that lactate levels can affect coagulation or platelet aggregation in all of these metabolites. Most elderly patients often have hidden blood loss after hip fracture^24^. Acute blood loss leads to decreased cardiac output, tachycardia, hypotension, and inadequate organ perfusion, which interferes with aerobic metabolism, and increased anaerobic metabolism leads to the production of lactic acid and metabolic acidosis. In addition, an increase or decrease in pH value can lead to disturbances in the internal environment. A hypoxic microenvironment at the fracture site is created by reduced or blocked blood supply, and anaerobic enzymes are enhanced, resulting in lactic acid accumulation, thus creating an acidic microenvironment with a low pH value at the fracture site^25,26^. Elevated lactate levels are thought to be associated with enhanced glycolysis in erythrocytes within the thrombus. A study by Maekawa et al.^27^ found lactate was the most abundant metabolite in the thrombus, with levels at least five times higher than those in venous blood. As Crowell et al. demonstrated, lactic acid stimulated whole blood clotting and impeded platelet aggregation to some extent. As the pH decreased, lactic acid infusion was significantly associated with a reduction in whole blood clotting time in vitro and in vivo^28^. In addition, the acid–base imbalance can damage the vascular endothelium, and damaged endothelial cells can release tissue factors, which activate the blood barrier system, reduce fibrinolytic activity, and enhance platelet adhesion and aggregation. The potential impact of an acidic environment with low pH values on cell function is essential^29^. A decrease in blood pH can lead to an increase in the enzymatic activity of coagulation factors, a reduction in the anticoagulant activity of heparin, and an increase in the ability to aggregate platelets, leading to hypercoagulation of the blood and, ultimately, DVT. Therefore, we speculate that the correlation between pH and DVT is local lactic acid accumulation in trauma caused by blood loss, which changes the pH value of the internal environment and affects the normal metabolic environment through several pathways, leading to the formation of DVT. However, this hypothesis should be tested in future studies.

In the current study, we performed univariate analysis to derive a more reliable relationship between admission pH and DVT and identified confounding factors for DVT formation. Sex, fracture classification, multiple injuries, time to operation, dementia, gastritis and activated partial thromboplastin time were identified as confounding factors. Multifactorial logistic regression was performed after further adjustment for confounding variables, and the results were found to be statistically significant when pH was used as a continuous variable but not in the trend test. A curvilinear relationship between pH and DVT was inferred using curve fitting. A pH value of 7.39 was found to be the inflection point in the curve.

This study is the first to explore the relationship between pH at admission and DVT formation. A curvilinear relationship was found between the two factors for the first time. Regarding other blood data (D-dimer, fibrinogen, activated partial thromboplastin time, prothrombin time, thrombin time, albumin, hemoglobin, total cholesterol, triglycerides), a meta-analysis reported no significant differences in these parameters^30^. This underscores the importance of pH as a predictor of DVT.

This study has some limitations. First, this retrospective study explored the relationship between admission pH and DVT formation. However, this study could not determine a causal relationship between these two factors. Second, our study population was from the largest trauma center in Northwest China. There may have been some bias in the selection of the study population due to region, ethnicity, and lifestyle habits. This results in findings that cannot be generalized to the entire population. All of these shortcomings need to be further confirmed in future studies.

In conclusion, the pH value is nonlinearly associated with preoperative DVT in geriatric patients with hip fractures and could be considered a predictor of the risk of DVT.

## Data Availability

The data was implemented by Xi’an Honghui Hospital. According to relevant regulations, the data could not be shared, but could request from correspondence author.

## Abbreviations

aCCI: Adjusted charlson comorbidity index
CI: Confidence interval
DVT: Deep vein thrombosis
OR: Odds ratio
PSM: Propensity score matching
